# Acute Encephalopathy and Cardiac Tamponade as Initial Presentations of High-Grade B-Cell Lymphoma

**DOI:** 10.7759/cureus.113643

**Published:** 2026-07-30

**Authors:** Aisha Krieger-Hussain, Zaineb Ahmad, Lejla Hodzic, Besher Kabak

**Affiliations:** 1 Emergency Medicine, Touro College of Osteopathic Medicine, Nyack, USA; 2 Internal Medicine, Touro College of Osteopathic Medicine, Middletown, USA; 3 Dermatology, Kansas City University of Medicine and Biosciences, Nyack, USA; 4 Critical Care, Montefiore Nyack Hospital, Nyack, USA

**Keywords:** acute encephalopathy, cardiac tamponade, high-grade b-cell lymphoma, malignant pericardial effusion, pericardial window, pericardiocentesis, pleural effusion

## Abstract

Cardiac tamponade is a life-threatening emergency caused by the accumulation of pericardial fluid, resulting in impaired cardiac filling. Although malignancy is a recognized cause of pericardial effusion, cardiac tamponade is a rare initial presentation of high-grade B-cell lymphoma (HGBCL) and may pose a diagnostic challenge because of its nonspecific clinical manifestations.

A 67-year-old man with a history of hypertension, hyperlipidemia, and schizophrenia presented from a group home with acute altered mental status and dyspnea. Initial computed tomography of the head was negative for acute cerebrovascular pathology but incidentally demonstrated a large pericardial effusion and bilateral pleural effusions. Laboratory evaluation revealed severe hyponatremia, elevated B-type natriuretic peptide, and rhabdomyolysis. Transthoracic echocardiography confirmed a large circumferential pericardial effusion with findings concerning for cardiac tamponade, prompting emergent pericardiocentesis with drainage of 2.2 L of hemorrhagic fluid. Although he initially improved, his hospital course was complicated by hypoxemic respiratory failure requiring mechanical ventilation, persistent bilateral pleural effusions, and atrial arrhythmias. Cytologic examination of pleural fluid demonstrated HGBCL. The patient subsequently underwent a pericardial window for recurrent pericardial effusion, improved clinically, was successfully extubated, and was discharged with outpatient hematology follow-up to initiate systemic chemotherapy.

This case illustrates cardiac tamponade as a rare but potentially fatal initial manifestation of HGBCL. Recognition of hemorrhagic pericardial effusion as a possible presentation of occult hematologic malignancy is critical, as prompt pericardial decompression combined with timely diagnostic evaluation can facilitate early oncologic treatment and improve clinical outcomes

## Introduction

High-grade B-cell lymphomas (HGBCLs) are aggressive non-Hodgkin lymphomas that often present with advanced disease and extranodal involvement, most commonly involving the gastrointestinal tract, bone marrow, and central nervous system [1]. Cardiac involvement, however, is not considered a characteristic manifestation of HGBCL. Across malignant lymphomas, cardiac involvement, usually in the form of a metastatic tumor, has been reported in approximately 1%-2% of cases and is largely described in isolated case reports, with diffuse large B-cell lymphoma comprising the majority of reported histologic subtypes [2,3]. Malignant pericardial effusions typically present with progressive cardiopulmonary symptoms, including dyspnea and signs of heart failure. Cardiac tamponade is a life-threatening condition caused by fluid accumulation in the pericardial sac, leading to impaired filling and subsequent hemodynamic compromise. Acute cardiac tamponade as the initial presentation of lymphoma is rare, and presentations dominated by acute neurologic symptoms are particularly unusual. We report a case of HGBCL presenting with acute cardiac tamponade manifesting as altered mental status and hypoxemic respiratory failure, highlighting the diagnostic challenges of atypical presentations and the importance of maintaining a broad differential diagnosis in patients with unexplained cardiopulmonary and neurologic compromise.

## Case presentation

**Figure 1:**
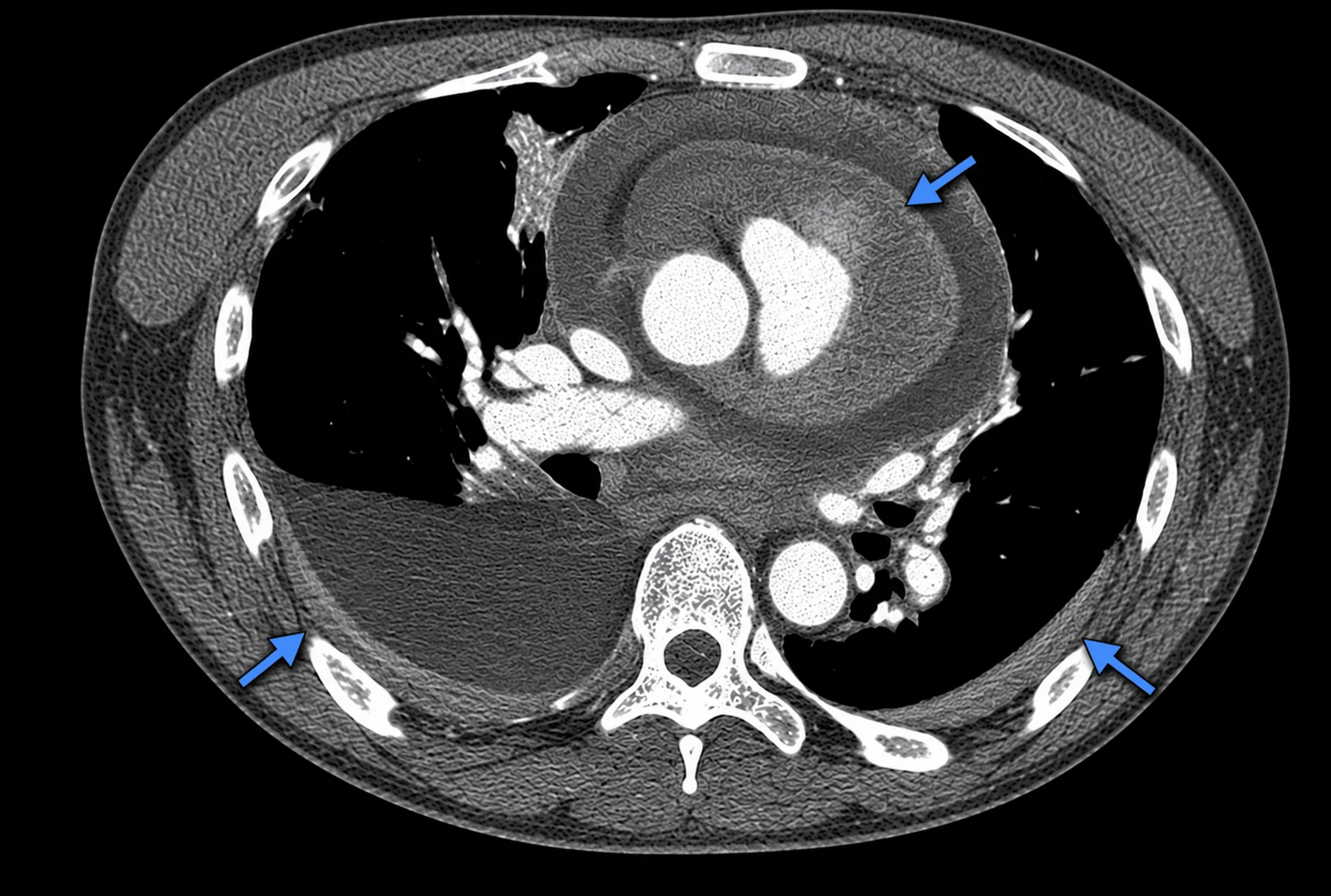
Computed tomography angiography (CTA) demonstrating incidental cardiopulmonary findings. The upper arrow indicates a large pericardial effusion, while the lower arrows indicate bilateral pleural effusions, greater on the right. CTA showed no large-vessel occlusion or hemodynamically significant stenosis. The pericardial effusion was incompletely imaged on this examination.

**Figure 2:**
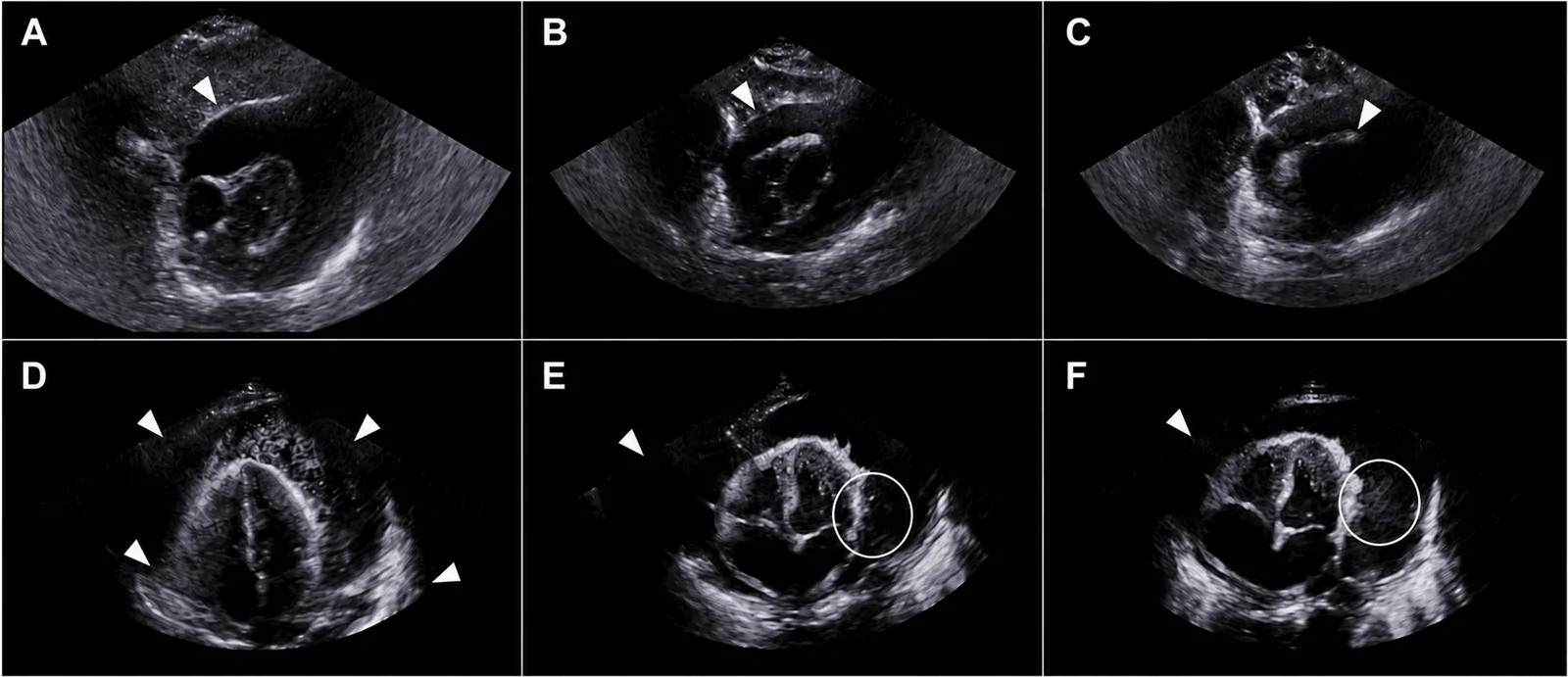
Transthoracic echocardiogram demonstrating a large pericardial effusion. Global left ventricular ejection fraction was approximately 55%. Diastology was indeterminate. Right ventricular cavity size appeared moderately dilated. Transthoracic echocardiography demonstrated a large circumferential pericardial effusion. Parasternal short-axis views (A-C) showed extensive pericardial fluid surrounding the left ventricle. Apical four-chamber views (D-F) confirmed the large circumferential effusion and demonstrated moderate right ventricular dilatation. Left ventricular size and systolic function were preserved, with an estimated ejection fraction of approximately 55% and no regional wall motion abnormalities. The inferior vena cava was dilated with less than 50% inspiratory collapse, and significant respiratory variation was present. Although these findings raised concern for evolving tamponade physiology, there was no echocardiographic evidence of right atrial or right ventricular collapse. A large pleural effusion was also noted.

A 67-year-old man with a history of hypertension, hyperlipidemia, and schizophrenia presented from a group home with acute altered mental status and dyspnea. Social history was notable for use of alcohol, tobacco, and recreational drugs; quantity, duration, frequency, and cessation dates unknown. He was last seen at baseline smoking a cigarette approximately two hours before presentation. On arrival, he was disoriented and tachypneic with jugular venous distention, diffuse adventitious lung sounds, and stable vitals. At this point, differentials included metabolic encephalopathy, respiratory infection, cerebral ischemia versus hemorrhage, among others. As per protocol for acute neurological symptoms, a stroke workup was initiated and was negative for ischemia or hemorrhage. However, computed tomography of the head/neck incidentally revealed a large pericardial effusion and bilateral pleural effusions (Figure 1). Laboratory studies were notable for severe hyponatremia, elevated B-type natriuretic peptide, rhabdomyolysis, lactic acidosis, and hypoxemia. These laboratory values are summarized in Table 1. Transthoracic echocardiography demonstrated a large pericardial effusion with features consistent with cardiac tamponade (Figure 2).

**Table 1:** Laboratory values at admission.

Laboratory parameter	Patient’s value	Reference value
Sodium	112 mmol/L	136-145 mmol/L
B-type natriuretic peptide	1325 pg/mL	<125 pg/mL
Creatinine phosphokinase	1515 U/L	39-308 U/L
Lactic acid	4.5 mmol/L	0.5-1.9 mmol/L

**Figure 3:**
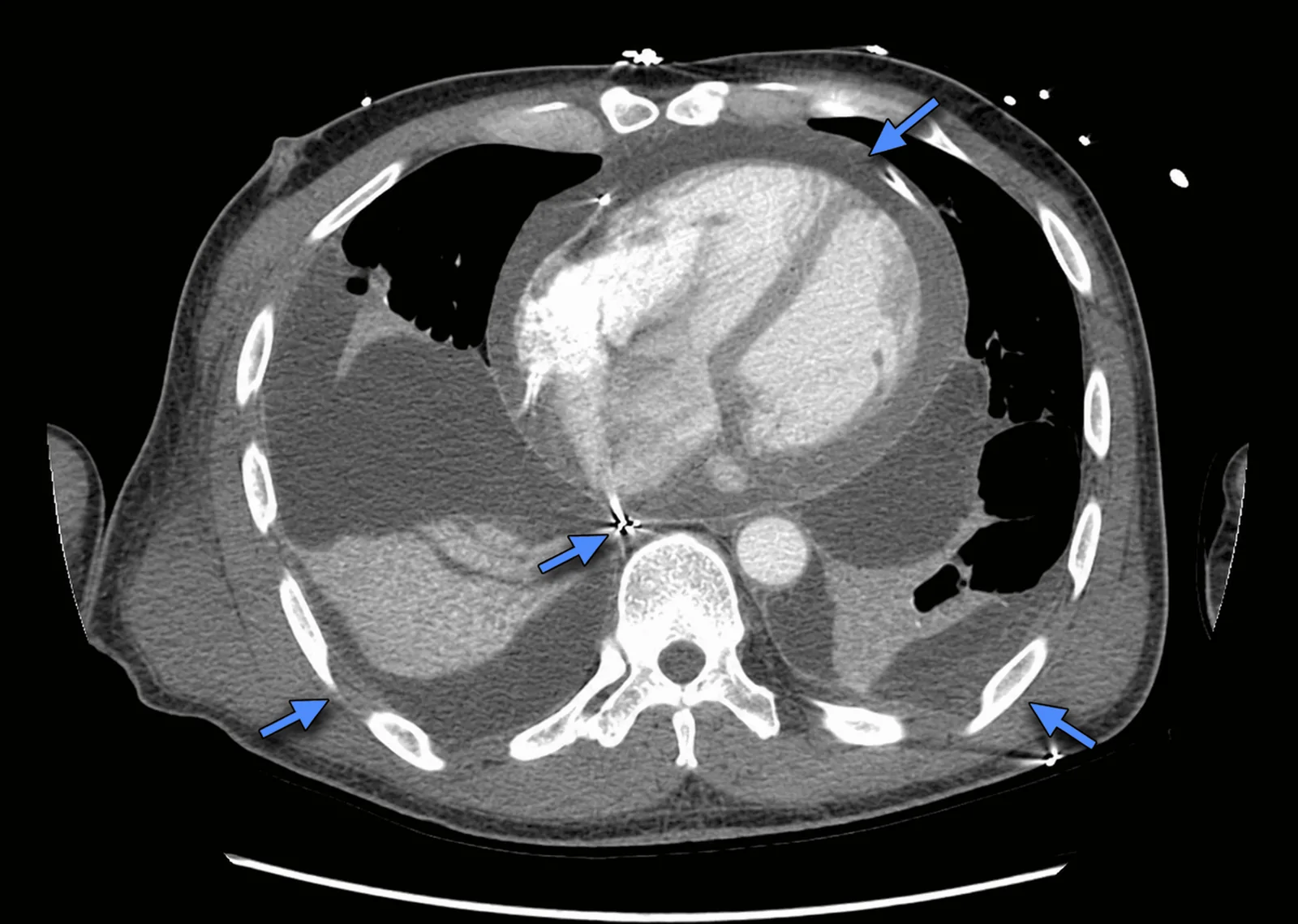
Computed tomography angiography (CTA) of the chest obtained after pericardiocentesis. The upper arrow indicates a residual moderate pericardial effusion. The central arrow indicates the pericardial drain in situ. The lower right and lower left arrows indicate persistent bilateral pleural effusions, including a moderate right pleural effusion and a small left pleural effusion, with associated partial collapse of the right and left lower lobes. Despite interval drainage, residual pericardial and pleural fluid collections remained. Underlying malignancy could not be excluded.

**Figure 4:**
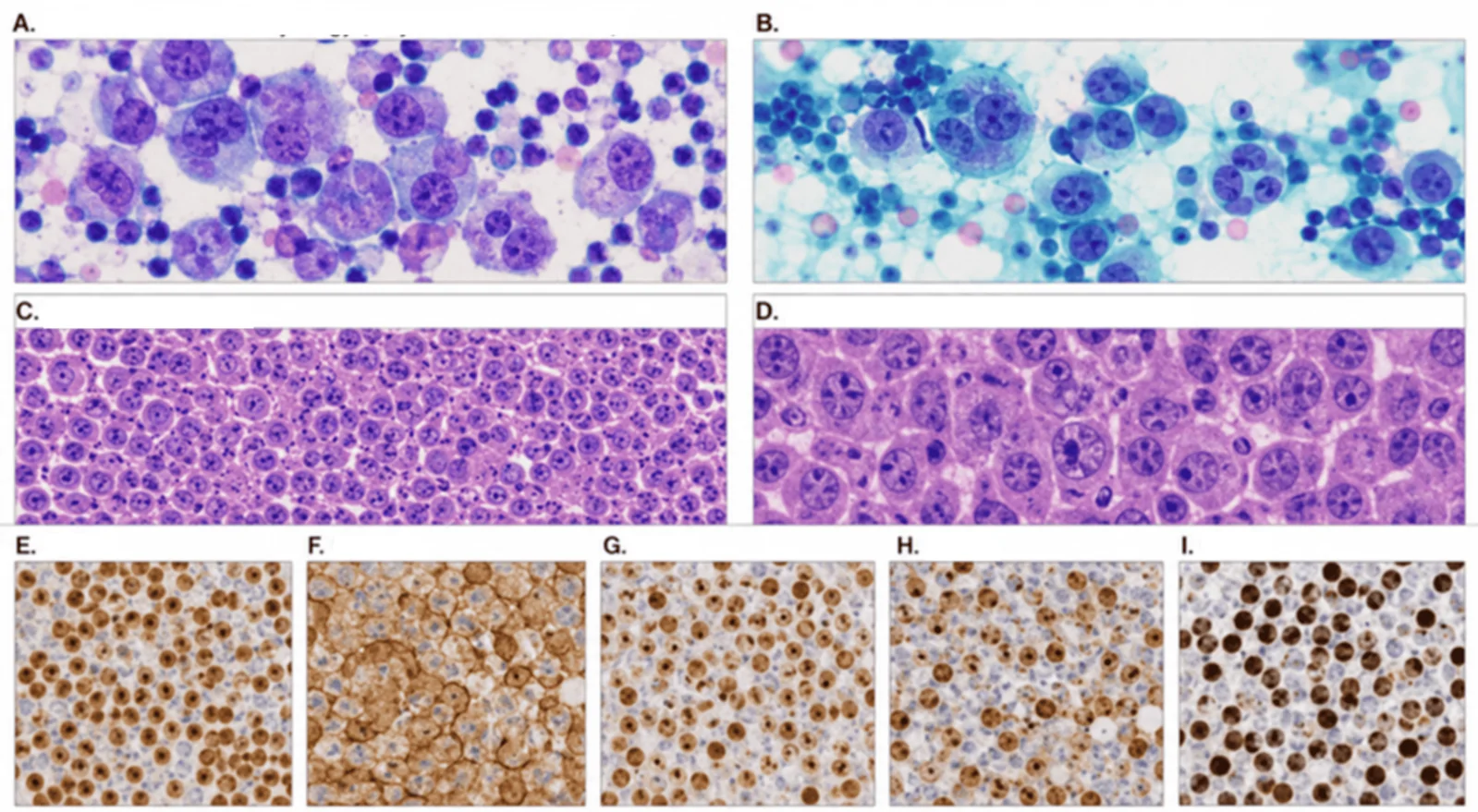
Cytologic analysis of pericardial and pleural fluid specimens demonstrating involvement by high-grade B-cell lymphoma. (A) Pericardial effusion cytology (May-Grünwald-Giemsa stain) demonstrating large atypical lymphoid cells with enlarged nuclei, vesicular chromatin, prominent nucleoli, and abundant cytoplasm in a background of reactive lymphocytes. (B) Pleural effusion cytology (Papanicolaou stain) showing scattered large neoplastic lymphoid cells with high nuclear-to-cytoplasmic ratios and conspicuous nucleoli. (C) Hematoxylin and eosin (H&E)-stained cell-block section demonstrating diffuse infiltration by large atypical lymphoid cells. (D) Higher-magnification H&E image highlighting marked nuclear pleomorphism, vesicular chromatin, and prominent nucleoli characteristic of aggressive B-cell lymphoma. (E) PAX5 immunohistochemistry showing strong nuclear staining in neoplastic cells, confirming B-cell lineage. (F) CD20 immunohistochemistry demonstrating membranous expression in the tumor cells. (G) BCL6 immunohistochemistry showing nuclear positivity. (H) MUM1 immunohistochemistry demonstrating nuclear expression consistent with an activated B-cell phenotype. (I) Ki-67 immunohistochemistry revealing a high proliferative index. Collectively, the cytomorphologic and immunophenotypic findings support the diagnosis of high-grade large B-cell lymphoma involving both the pericardial and pleural spaces.

The patient underwent emergent pericardiocentesis with drainage of 2.2 L of hemorrhagic fluid, resulting in transient clinical improvement. However, after admission to the medical intensive care unit, he slowly decompensated, and despite draining his effusions, his respiratory status unexpectedly declined. His hospital course was complicated by hypoxemic respiratory failure requiring intubation and mechanical ventilation. He underwent computed tomography angiography (CTA) of the chest, abdomen, and pelvis, which continued to show his effusions (Figure 3). Throughout his admission, he had repeated echocardiograms demonstrating the persistent, loculated pleural effusions and pericardial effusion. Cytologic analysis of both the pleural and pericardial fluids revealed high-grade B-cell lymphoma (Figure 4). Due to ongoing cardiopulmonary issues, including life-threatening arrhythmias, a pericardial window procedure was performed to drain additional fluid. The patient subsequently improved, was extubated, and discharged back to his facility with plans for outpatient chemotherapy following hematology-oncology evaluation.

## Discussion

Cardiac involvement of any type of cancer presents as a metastatic tumor, most commonly from cancers of the lung, esophagus, and breast. Cardiac involvement is rare in lymphoma overall, reported in only a small minority of cases, even among cohorts undergoing systematic echocardiographic evaluation [2]. The existing literature on cardiac lymphoma is dominated by diffuse large B-cell lymphoma and heterogeneous *malignant lymphoma *cohorts, in which cardiac involvement is infrequent and typically described through small case series or individual case reports [3]. In patients with Hodgkin's and non-Hodgkin's lymphoma who underwent autopsy, cardiac manifestation was found in 16% and 18% of patients, respectively [4]. In the cases that do exist, the majority of patients present with symptoms of congestive heart failure, such as shortness of breath, chest pain, and edema.

Malignant pericardial effusions typically develop gradually and present with progressive symptoms. These effusions can build up and put pressure on certain structures, causing cardiac tamponade. Cardiac tamponade as the first manifestation of HGBCL is exceptionally uncommon. Hemorrhagic pericardial effusions, as seen in this case, should raise suspicion for malignancy, particularly in the absence of trauma or procedural causes [5,6]. Cytologic evaluation remains a cornerstone of diagnosis; its sensitivity ranges from approximately 60% to 80%, and a negative result does not exclude malignancy [5,7]. Additional diagnostic modalities, including flow cytometry and pericardial biopsy, may be required in select cases [7].

A notable aspect of this case is the presentation with acute encephalopathy, mimicking a cerebrovascular event. Altered mental status is not a typical presenting symptom of pericardial effusion or tamponade. In this patient, encephalopathy was likely multifactorial, with contributions from severe hyponatremia, hypoxemia, rhabdomyolysis, and reduced cerebral perfusion secondary to tamponade physiology. Hyponatremia itself is a well-recognized cause of acute encephalopathy and may further complicate diagnostic evaluation in critically ill patients.

Additionally, the patient's clinical deterioration following pericardiocentesis was unexpected. In most cases, drainage of tamponade results in rapid hemodynamic and symptomatic improvement [8]. In this case, the deterioration was most likely due to Pericardial Decompression Syndrome (PDS). PDS is a rare condition marked by paradoxical hemodynamic worsening after large-volume pericardiocentesis. Symptoms appear immediately or within hours. Persistent or worsening respiratory failure after pericardiocentesis should prompt evaluation for additional underlying processes, including malignancy, persistent pleural effusions, or systemic inflammatory responses [6,9].

This case underscores the importance of maintaining a broad differential diagnosis in a patient presenting with unexplained cardiopulmonary compromise and altered mental status, particularly when the clinical course deviates from expected improvement following standard interventions. Early recognition of malignant causes of pericardial effusion is essential, as timely diagnosis can significantly impact management and prognosis.

## Conclusions

Cardiac tamponade may serve as a rare initial manifestation of aggressive malignancies such as HGBCL. This case highlights that tamponade can present atypically as acute encephalopathy, mimicking neurologic emergencies such as stroke. However, this patient's altered mental status was likely multifactorial due to their hemodynamic instability, electrolyte abnormalities, and lactic acidosis. Clinicians should maintain a broad differential diagnosis when evaluating patients with unexplained altered mental status and cardiopulmonary compromise. Furthermore, a lack of sustained improvement after pericardiocentesis should raise suspicion for an underlying malignant or systemic process. Early recognition and appropriate diagnostic evaluation, including cytologic analysis, are critical for timely diagnosis and management.
